# A Comparative Study of the Inhibitory Effect of Some Flavonoids and a Conjugate of Taxifolin with Glyoxylic Acid on the Oxidative Burst of Neutrophils

**DOI:** 10.3390/ijms242015068

**Published:** 2023-10-11

**Authors:** Victoria S. Shubina, Victoria I. Kozina, Yuri V. Shatalin

**Affiliations:** Institute of Theoretical and Experimental Biophysics, Russian Academy of Sciences, Institutskaya 3, 142290 Pushchino, Russia; ernie.nike@yandex.ru

**Keywords:** flavonoids, glyoxylate-taxifolin condensation product, neutrophils, oxidative burst, reactive oxygen species

## Abstract

During the storage, processing, and digestion of flavonoid-rich foods and beverages, a condensation of flavonoids with toxic carbonyl compounds occurs. The effect of the resulting products on cells remains largely unknown. The aim of the present study was to evaluate the effects of quercetin, taxifolin, catechin, eriodictyol, hesperetin, naringenin, and a condensation product of taxifolin with glyoxylic acid on the oxidative burst of neutrophils. It was found that the flavonoids and the condensation product inhibited the total production of ROS. Flavonoids decreased both the intra and extracellular ROS production. The condensation product had no effect on intracellular ROS production but effectively inhibited the extracellular production of ROS. Thus, the condensation of flavonoids with toxic carbonyl compounds may lead to the formation of compounds exhibiting potent inhibitory effects on the oxidative burst of neutrophils. The data also suggest that, during these reactions, the influence of a fraction of flavonoids and their polyphenolic derivatives on cellular functions may change. On the whole, the results of the study provide a better understanding of the effects of polyphenols on human health. In addition, these results reveal the structure–activity relationship of these polyphenols and may be useful in a search for new therapeutic agents against diseases associated with oxidative stress.

## 1. Introduction

**Figure 1:**
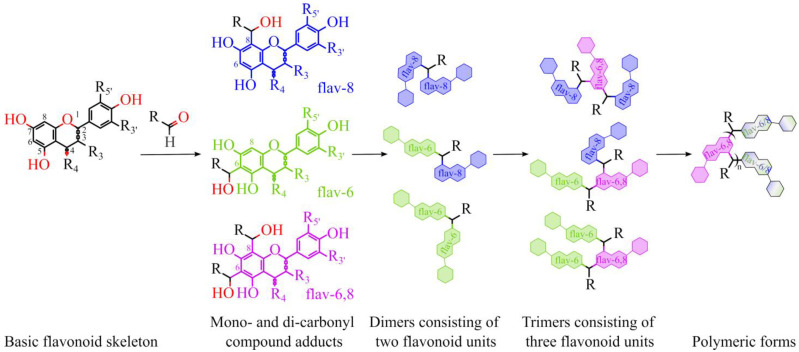
Schematic representation of the proposed mechanism for the condensation of some flavonoids and carbonyl compounds. Some flavonoids for which the formation of the adducts was found: epigallocatechin gallate [41,42], epicatechin [43,44], catechin [29,43], naringenin [45], quercetin [32,46], rutin [36,37], taxifolin [47], kaempferol [41,48], myricetin [49].

Polyphenols are secondary metabolites widely distributed in the Plant Kingdom. They are involved in various physiological functions of plants, including the protection against unfavorable environmental factors. Along with beneficial effects on plants, many polyphenols are considered compounds that can positively influence human health. The consumption of polyphenol-rich foods and beverages may be associated with a reduction of the risk of cardiovascular [1,2,3], neurodegenerative [4,5,6,7], and some other diseases [8,9]. Polyphenols show a broad spectrum of biological activities, including antioxidant [10,11], anti-inflammatory [12], antibacterial [13], antiplatelet [14,15,16,17], antihypertensive [18,19,20,21], and anticarcinogenic activities [22]. Polyphenols are highly varied in structure, but all of them contain at least one benzene ring bearing several hydroxyl groups [23]. Depending on the number of benzene rings and the structural elements that bind these rings to one another [24], polyphenols can be divided into several classes, from relatively simple, such as phenolic acids, to polymerized molecules of relatively high molecular weight, such as condensed tannins [24,25]. Minimally processed fresh plant foods and beverages contain relatively high levels of polyphenols [23,25,26]. However, during storage, aging, and processing, polyphenols undergo many modifications [23,27]. In particular, polyphenols trap reactive carbonyl compounds, such as methylglyoxal (MGO), glyoxylic acid, acetaldehyde, and furfurol, which results in the generation of monomeric, oligomeric, and polymeric adducts (Figure 1) [28,29,30,31,32,33,34,35]. Some of these, e.g., rutin–MGO adducts, have already been identified in commercial food products [36,37]. Presumably, the interaction of polyphenols with carbonyl compounds contributes to their protective effect against lipid oxidation-induced damage to food components [23,38,39,40].

After entering the gastrointestinal tract (GIT), polyphenols either remain in their native form or undergo further transformations [50]. Both parent and modified polyphenols can be absorbed and have systemic effects or/and can remain in the GIT and exert local actions [50]. In the latter case, the effects of polyphenols can be perceptible since their concentration in the GIT can reach relatively high values [25,50,51]. In particular, polyphenols can exert antioxidant action due to interactions with reactive oxygen species (ROS) and transition metal ions, or they can directly react with toxic compounds formed during the preparation and digestion of food. To date, there is substantial evidence that the consumption of foods containing partially oxidized lipids increases the level of toxic hydroperoxides and malonic aldehyde in the stomach and, after their absorption, in the blood plasma. The addition of phenol-rich foods or beverages to the diet substantially reduces or completely prevents the accumulation of these toxic compounds [52,53,54,55,56]. Recent studies have shown that flavonoids form adducts with carbonyl compounds in vivo [36,37]. In particular, it was found that genistein [57,58], myricetin [49], epicatechin, and (6)-shogaol [59] efficiently trapped MGO and generated mono- and di-MGO adducts in mice. Rutin–MGO adducts were also identified in rats [36,37]. In general, the available data suggest that the consumption of polyphenols may prevent harmful effects on human health induced by reactive carbonyl species [36,52,53,60].

Although it is common knowledge that polyphenols react with toxic carbonyl compounds during storage, aging, processing, and digestion of polyphenol-rich foods and beverages, the properties of the resulting compounds and the impact of these reactions on the properties of polyphenolic fractions are little studied, mainly, due to challenges in characterization of these reactions as well as identification and isolation of polyphenol adducts [23]. The effect of the resulting products on cells also remains largely unknown.

Our previous results indicate that a condensation product of taxifolin with glyoxylic acid (DfTf) possesses high antioxidant activity against ROS present in the aqueous phase. In particular, DfTf is more effective in scavenging hydrogen peroxide than the parent flavonoid taxifolin and its structural analogs quercetin, catechin, eriodictyol, hesperetin, and naringenin [47,61]. On the other hand, DfTf inhibits lipid peroxidation (LPO) worse than quercetin, eriodictyol, and catechin, suggesting its lower efficiency toward ROS generated in a biphasic system. Probably, this is related, at least partially, to the physicochemical properties of this polyphenol. It was shown that DfTf is the most hydrophilic among all compounds tested [47]. It can be assumed that more hydrophobic quercetin, catechin, and eriodictyol accumulate in the lipid in larger quantities than hydrophilic DfTf, which enhances the protective effect of these compounds against lipid peroxidation. However, any significant difference between the inhibitory effects of DfTf and taxifolin was not found. In general, our data indicate that the properties of parent polyphenols and the condensation products of flavonoids with toxic carbonyl compounds, as well as the conditions under which they show the maximum antioxidant activity, can vary [47,61].

Taking the aforementioned into account, it seems relevant to compare the effects of polyphenols and their conjugates with carbonyl compounds on ROS production by specialized cells such as neutrophils.

Neutrophils are the most abundant leukocytes in the blood. These cells are among the first to be recruited to the sites of infection and inflammation, where they eradicate invading pathogens and initiate and sustain the inflammatory process [62]. The production of ROS by neutrophils plays a key role in microbial killing. In addition, ROS can act as signaling molecules in the inflammatory process. However, excessive production of ROS can lead to inflammatory tissue damage [63,64,65,66]. In particular, there is evidence indicating that neutrophils play an important role in the inflammation of the GIT, the site where, as was mentioned above, the concentration of polyphenols reaches relatively high values [66,67]. It may be suggested that polyphenols, under certain conditions, can modulate the functional responses of neutrophils and influence the level of ROS generated by these cells.

**Figure 2:**
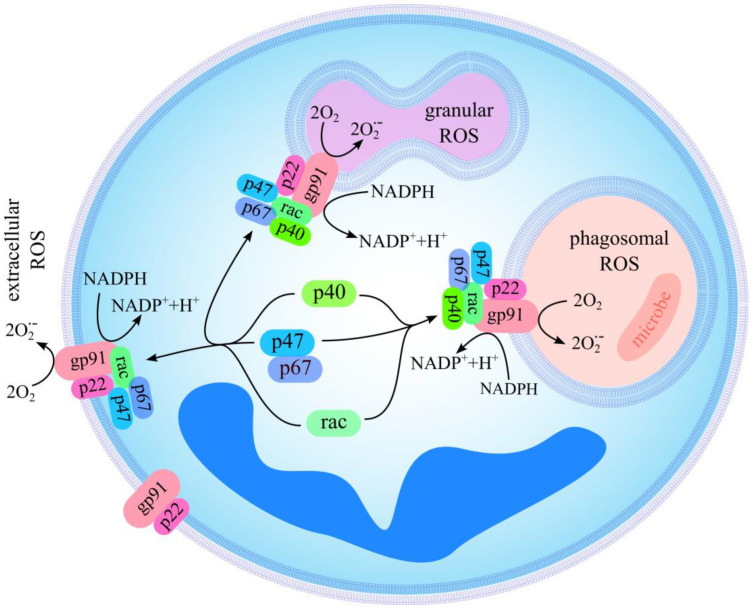
Assembly of NADPH oxidase and ROS production.

Neutrophils produce ROS via the multisubunit enzyme complex called NADPH oxidase (NOX2) (Figure 2). Two subunits of NOX2, gp91*^phox^* and p22*^phox^*, are localized mainly in the membrane of specific granules and in the cytoplasmic membrane. Together, they comprise the large heterodimeric subunit flavocytochrome b_558_ (cyt b_558_) [68,69]. In resting neutrophils, the subunits p40*^phox^*, p47*^phox^*, and p67*^phox^* are localized in the cytosol. Upon activation, they, together with Rac-GTPase, are translocated to the membrane and associate with cyt b_558_ to form active oxidase [68,69]. The activated complex transfers electrons across the membrane from cytosolic NADPH to O_2_ to form superoxide anion (O_2_^•−^), which spontaneously dismutates to hydrogen peroxide (Figure 2). O_2_^•−^ and H_2_O_2_ can subsequently be transformed into other ROS.

The activation of NADPH oxidase at the plasma membrane causes an extracellular release of ROS, whereas its activation at the membrane of a granule leads to the production of ROS inside the cell (Figure 2). The predicted site for intracellular ROS production would typically be phagolysosomes [69]. However, intracellular ROS can also be produced if phagosomes do not form [69,70]. In particular, this occurs after the stimulation of neutrophils with phorbol 12-myristate 13-acetate (PMA) [69,71]. It can be suggested that intracellular ROS can be produced in a compartment formed after the fusion of heterotypic granules [69,72]. The activation of these two pools of NADPH oxidase (in the plasma membrane and in the membrane of granules) proceeds via slightly different signaling pathways [69,73], suggesting that the regulation of activity of the complex can also be different for these two pools [69]. In particular, the cytoplasmic subunit p40*^phox^* plays a key role in the production of intracellular ROS but is not dispensable for the production of extracellular ROS. Taking into account that enhanced production of extracellular ROS by neutrophils at the site of inflammation causes tissue injury, whereas the production of intracellular ROS is involved in microbial killing and regulation of functions of neutrophils, the identification of agents and the signaling pathways that specifically control extracellular, but not intracellular ROS production, is of great therapeutic interest [74,75].

**Figure 3:**
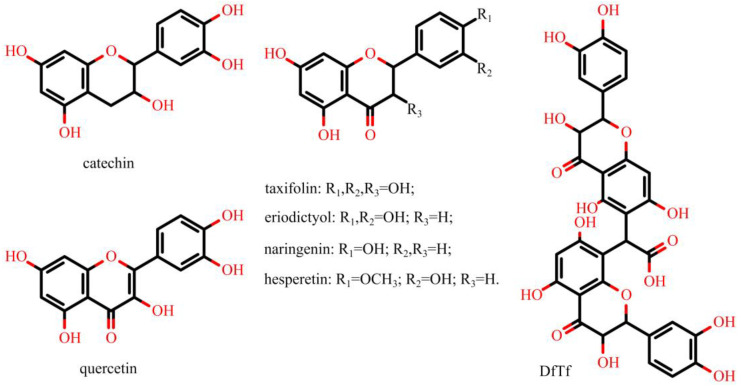
Structures of polyphenols being tested. The product of the condensation of taxifolin with glyoxylic acid is designated as DfTf.

The aim of the present work was a comparative study of the effects of six structurally similar flavonoids (quercetin, taxifolin, catechin, eriodictyol, hesperetin, naringenin) and a condensation product of taxifolin with glyoxylic acid (Figure 3) on the total, intra and extracellular production of ROS.

## 2. Results

Intra and extracellular ROS production by neutrophils was measured by the method of luminol-dependent chemiluminescence (LCL). Luminol can penetrate into cells and react with ROS generated both inside and outside of cells [76]. Intracellular ROS were detected by LCL in the presence of membrane-impermeable enzymes, superoxide dismutase (SOD), and catalase, which remove extracellularly released O_2_^•−^ and H_2_O_2_. Extracellular ROS were also detected by CL using isoluminol, a membrane-impermeable isomer of luminol, as a dye [76]. Luminol and isoluminol-dependent chemiluminescence assays can also be used for the estimation of the antioxidant activity of compounds in cell-free systems [77,78]. Taking this into account, the question of how polyphenols behave in these systems was of special interest.

### 2.1. Hydrogen Peroxide Scavenging Activity

We have previously shown that DfTf, quercetin, taxifolin, catechin, and eriodictyol exhibited higher antioxidant activity in a cell-free system containing luminol, horseradish peroxidase (HRP), and H_2_O_2_ than hesperetin and naringenin [61]; i.e., compounds containing a catechol moiety appeared to be more active. DfTf, which has two catechol moieties, exhibited the highest scavenging activity toward hydrogen peroxide [61].

**Figure 4:**
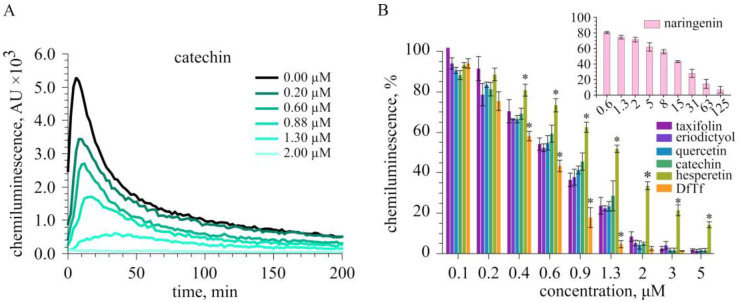
Effect of polyphenols on isoluminol-dependent chemiluminescence in a cell-free system. (**A**) The time courses of chemiluminescence in the control and in the presence of different concentrations of catechin. (**B**) Comparison of the effect of polyphenols on integral chemiluminescence. The values are the means ± standard deviation. The data were analyzed using one-way ANOVA followed by Tukey’s test. * *p* < 0.05 compared with other compounds.

In the present work, we examined the hydrogen peroxide-scavenging activity of polyphenols in a cell free-system containing isoluminol, HRP, and H_2_O_2_. As an example, Figure 4A presents the time courses of isoluminol-dependent chemiluminescence (ICL) in the control and in the presence of different concentrations of catechin. Figure 4B shows the effect of polyphenols on integral ICL. It can be seen that ICL decreased in a dose-dependent manner in the presence of all polyphenols tested. The IC_50_ values (the concentration of a compound at which the integral ICL decreases by 50%) are given in Table 1. Similar to the luminol-HRP-H_2_O_2_ system, catechol-containing polyphenols inhibited ICL to a greater extent than hesperetin and naringenin. DfTf showed the highest antioxidant activity. For comparison, the IC_50_ values previously obtained using a luminol-HRP-H_2_O_2_ system [61] are also presented in Table 1.

### 2.2. Effect of Polyphenols on the Total Production of ROS

**Figure 5:**
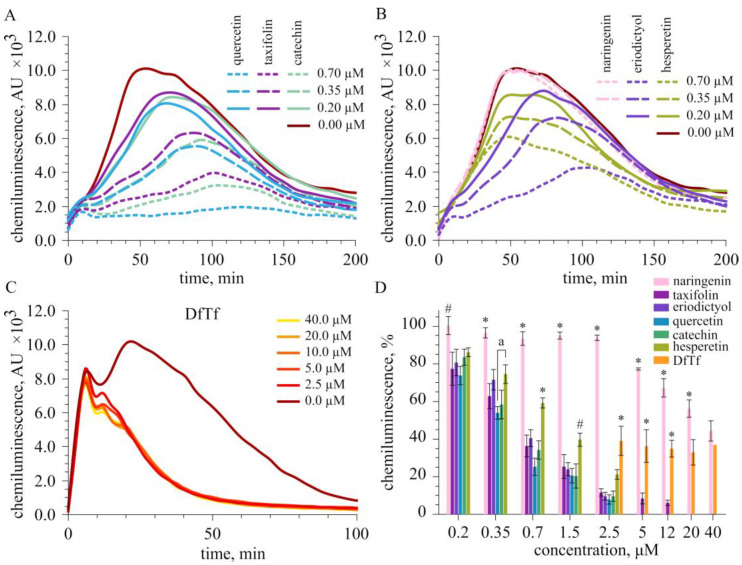
Effect of polyphenols on luminol-dependent chemiluminescence of neutrophils stimulated with PMA. (**A**–**C**) The time courses of luminol-dependent chemiluminescence of neutrophils in the control and the presence of different concentrations of polyphenols. (**D**) Comparison of the effect of polyphenols on integral chemiluminescence. The results were expressed as the mean ± standard deviation. The data were analyzed using one-way ANOVA followed by Tukey’s test. * *p* < 0.001 and # *p* < 0.05 compared with other compounds; a significant differences between the groups, *p* < 0.001.

It was found that flavonoids dose-dependently decreased luminol-dependent chemiluminescence (LCL) of neutrophils stimulated with PMA (Figure 5). The IC_50_ values are presented in Table 2. As seen from Figure 5 and Table 2, flavonoids containing a catechol fragment in their structure (quercetin, taxifolin, catechin, and eriodictyol) effectively inhibited the LCL response. These compounds exhibited similar inhibitory activity. Hesperetin was a less potent inhibitor of LCL than catechol-containing flavonoids, and naringenin produced the least inhibitory effect.

DfTf also decreased the LCL of neutrophils. Although DfTf contains two catechol moieties in the structure, it inhibited LCL to a lesser extent than the catechol-containing flavonoids and hesperetin (Figure 5). Interestingly, the decrease in LCL was slightly dependent on the concentration of DfTf in the concentration range from 2.5 to 40 µM (Figure 5C). In this case, the dose-response cannot be captured with the optimization of a standard Hill model [79]. Therefore, the IC_50_ value was not determined. We hypothesized that this effect may be due to the selective inhibition of ROS formation inside and outside the cells. Therefore, at the next stage, we examined the effect of polyphenols tested on the extra- and intracellular part of a CL signal.

### 2.3. Effect of Polyphenols on Extracellular and Intracellular ROS Production

**Figure 6:**
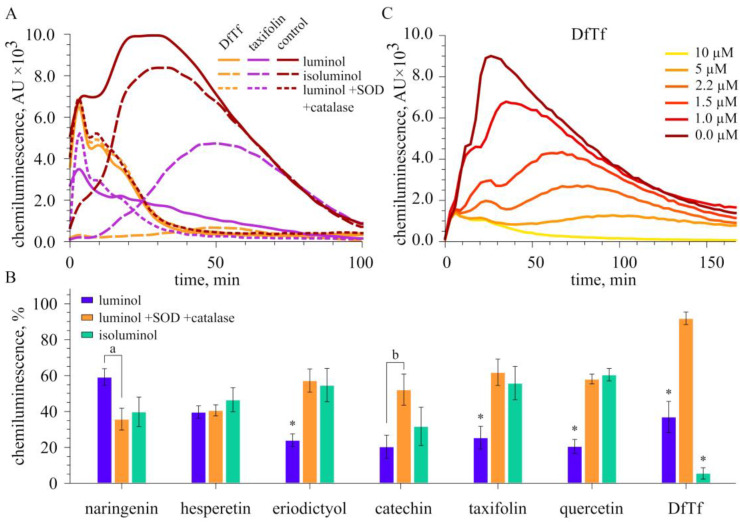
Effect of polyphenols on chemiluminescence of neutrophils stimulated with PMA. (**A**) The time courses of luminol and isoluminol-dependent chemiluminescence of neutrophils in the control and in the presence of taxifolin and DfTf. The concentration of taxifolin was 1.5 μM, and the concentration of DfTf was 5 μM. (**B**) Comparison of the effect of polyphenols on total, extra, and intracellular chemiluminescence. The concentration of polyphenols was 1.5 μM, except for the concentration of naringenin and DfTf, which was 20 and 5 μM, respectively. The results were expressed as the mean ± standard deviation. The data were analyzed using one-way ANOVA followed by Tukey’s test. * *p* < 0.005 compared with other groups (luminol + SOD + catalase; isoluminol); a significant differences between the groups, *p* < 0.02; b significant differences between the groups, *p* < 0.005. (**C**) The time courses of isoluminol-dependent chemiluminescence of neutrophils in the control and in the presence of different concentrations of DfTf.

Figure 6 shows the time courses of luminol and isoluminol-dependent CL of neutrophils stimulated with PMA in the presence and absence of taxifolin and its conjugate DfTf. It can be seen that taxifolin decreased both the intra and extracellular CL response of neutrophils. Similar results were obtained for the other flavonoids tested (Supplementary Figures S1 and S2). Both inside and outside the cells, the flavonoids showed comparable inhibitory activity. Catechol-containing flavonoids and hesperetin were more effective than naringenin (Figure 6).

Interestingly, taxifolin, eriodictyol, quercetin, and catechin decreased LCL to a greater extent than ICL, and LCL was recorded in the presence of SOD and catalase (Figure 6). This effect was not revealed for hesperetin and naringenin. Moreover, naringenin more effectively inhibited LCL in the presence of SOD and catalase (intracellular CL).

A completely different situation was observed in the case of DfTf. The time courses of LCL in the presence of DfTf (in the concentration range from 2.5 to 40 μM) were similar regardless of whether SOD and catalase were present in the system or not. Moreover, these time courses of LCL responses were similar to those observed in a system containing luminol, SOD, and catalase in the absence of any polyphenols (control). In all cases mentioned above, the integral LCL values were also comparable (Figure 6).

Thus, these data indicate that DfTf does not significantly affect the intracellular LCL response of neutrophils. At the same time, DfTf dose-dependently inhibits the extracellular ICL of neutrophils, its inhibitory activity being comparable with that of quercetin, taxifolin, eriodictyol, catechin, and hesperetin (Table 3). On the whole, the data suggest that DfTf does not interfere with the signaling pathways involved in intracellular ROS production by PMA-stimulated neutrophils when the elimination of extracellular ROS occurs. Based on these findings, it can also be assumed that this polyphenol selectively modulates neutrophil responses.

## 3. Discussion

The results of the study indicate that DfTf, eriodictyol, quercetin, taxifolin, and catechin exhibit high antioxidant activity in a system containing isoluminol, HRP, and H_2_O_2_. Previously, we have obtained similar results in a system containing luminol, HRP, and H_2_O_2_ [61]. These compounds contain in their structure hydroxyl groups located in the B ring in the *ortho*-position relative to each other (the catechol fragment) [61]. Two other polyphenols, hesperetin, which has a methoxy substituent at the 4′-position, and naringenin, which lacks a hydroxyl group at the 3′-position, were less effective as antioxidants in these two systems [61]. These results are in good agreement with the literature data, according to which the presence of *ortho*-dihydroxy groups in the B ring is an important structural feature of polyphenols showing high antioxidant activity [10,11]. In particular, it was shown that quercetin, catechin, and taxifolin were more active in scavenging the radicals generated in the aqueous phase (ABTS and DPPH radicals) than hesperetin and naringenin [10,11].

DfTf contains two catechol fragments and exhibits higher hydrogen peroxide- scavenging activity than the parent flavonoid taxifolin and its structural analogs (quercetin, eriodictyol, catechin, hesperetin, and naringenin) [47,61]. Although the properties of the products of condensation and polymerization of flavonoids remain poorly studied [23,80,81], there is evidence that these compounds exhibit high antioxidant activity, which, in many cases, exceeds that of parent flavonoids [81,82,83,84,85,86]. In particular, the polycondensates of catechin with acetaldehyde and poly(rutin) were shown to exhibit a stronger scavenging activity towards superoxide-anion and stronger inhibitory effects on the peroxidation of human low-density lipoproteins than the monomeric form of flavonoids [82,83,84]. Polymeric quercetin more effectively reduced DPPH radicals than monomeric quercetin [87]. Oligomerized epigallocatechin gallate (EGCG) exhibited higher superoxide-scavenging activity than its monomeric form [88]. Proanthocyanidins found in various plants showed potent antioxidant activity, which positively correlated with the degree of their polymerization [89,90,91,92]. Adducts formed by polyphenols such as quercetin and propyl gallate with methylglyoxal (mono-and di-MGO quercetin adducts and mono-MGO propyl gallate adduct) retained the ability to scavenge the DPPH radical and carbonyl compounds characteristic of parent flavonoids [32,93].

Thus, the condensation of flavonoids with toxic carbonyl compounds leads to the elimination of the latter and the formation of adducts showing high antioxidant activity.

Our previous results indicated that DfTf retains the ability to inhibit lipid peroxidation (LPO); however, it inhibits LPO worse than quercetin, catechin, and eriodictyol [61], suggesting that this polyphenol is less effective in scavenging ROS generated in a biphasic system. It can be assumed that this is related, at least partially, to the physicochemical properties of this polyphenol. DfTf is the most hydrophilic among all compounds tested [61] and accumulates in the lipid phase to a lesser extent than quercetin, catechin, and eriodictyol (Table 4). Probably, the accumulation of strong antioxidants in the lipid phase contributes to a more effective protection of lipids against oxidation. At the same time, the capacity of DfTf to inhibit LPO was comparable with that of taxifolin.

On the whole, our data indicate that the efficacy of flavonoids and their adducts as antioxidants can vary in different systems and under different environmental conditions.

Little has been reported regarding the potential effects of newly formed adducts on the cells of the body. Recently, data indicating that the formation of adducts of rutin with methylglyoxal remarkably reduced the toxicity of this aldehyde in GES-1, HUVEC, and PC-12 cell lines have been published [36,37]. In turn, the adducts themselves displayed a much lower toxicity, which was comparable with that of rutin [37]. Here, we estimated for the first time the influence of a conjugate of taxifolin with glyoxylic acid on the level of ROS generated by neutrophils stimulated with PMA and compared the effect of DfTf with that of taxifolin and its structural analogs.

It was found that all flavonoids decreased the LCL response of PMA-stimulated neutrophils (total ROS production) in a concentration-dependent manner. Catechol-containing flavonoids were more effective than the others and showed comparable inhibitory activity. These results are in agreement with the data available in the literature, in particular with the data of Ribeiro and coauthors [98,99]. The authors studied the impact of 24 flavonoids on the oxidative burst of human neutrophils using different detection probes, including luminol, lucigenin, amplex red, and APF. Their results indicate that, in all cases, the presence of a catechol group is essential for the manifestation of good activity. Among the compounds tested were quercetin, taxifolin, eriodictyol, and naringenin. It was shown that the oxidation of luminol by ROS generated by PMA-stimulated neutrophils was more effective in the presence of eriodictyol than in the presence of the other flavanones (taxifolin and naringenin) [98]. The flavonol quercetin was more effective than taxifolin and naringenin [98].

Our data indicate that DfTf is also an effective inhibitor of LCL. However, despite the fact that DfTf contains two catechol groups in the structure, it reduced the LCL response to a lesser extent than catechol-containing flavonoids and hesperetin. Interestingly, the decrease in the LCL was slightly dependent on the concentration of DfTf in the concentration range from 2.5 to 40 μM. As mentioned above, the effect of the adducts of flavonoids with carbonyl compounds on the production of ROS by neutrophils has not yet been studied. However, there are fragmentary data in the literature on the effects of oligomeric forms of flavonoids found in various plants on the oxidative burst of neutrophils [100,101]. In particular, Czerwińska and coauthors showed that procyanidins from small-leaved lime flowers (*Tilia cordata* Mill.) strongly inhibited ROS production by fMLP-stimulated neutrophils [101]. These compounds were more effective than epicatechin and quercetin. The inhibitory activity of procyanidins positively correlated with the number of epicatechin units in their structure [101]. Arwa and coauthors found that biflavonoids procyanidin, fukugetin, amentoflavone, and podocarpusflavone are effective inhibitors of the oxidative burst of PMA-stimulated neutrophils. The activity of these compounds was similar to that of quercetin [100].

Thus, our results and the literature data indicate that polyphenols containing two or more units of flavonoids can exhibit strong inhibitory activity against an oxidative burst of neutrophils. Most likely, the different effectiveness of these compounds may be due to differences in the mechanisms of their action [99,102].

Our further experiments showed that the flavonoids reduce both intra and extracellular ROS production by PMA-stimulated neutrophils. These compounds had a comparable inhibitory effect both inside and outside the cells. DfTf had no effect on intracellular ROS production but effectively decreased the extracellular level of ROS. It is significant that some catechol-containing compounds (taxifolin, eriodictyol, and quercetin) inhibited the total ROS production to a greater extent than the intra or extracellular production of ROS. This effect was not revealed for hesperetin and naringenin. Naringenin more effectively inhibited intracellular ROS production. Taking into account that hesperetin and naringenin are the most lipophilic compounds among all compounds tested (Table 4), it may be suggested that the lipophilicity of the compounds contributes to the effects mentioned above. It is known that the ability of compounds to incorporate into the lipid bilayer and the passive diffusion of compounds into cells depends on their lipophilicity. Presumably, the compounds studied have different intracellular accessibility, which can explain, at least partially, their different effectiveness.

This hypothesis was earlier proposed by Vuotto and coauthors [103], who showed that lipophilic catechins containing a hydrophobic propionyl or a valeryl group at the third position more intensively inhibited the intracellular CL response of PMA-stimulated neutrophils than native catechin. At the same time, all compounds similarly inhibited extracellular CL and showed similar antioxidant activity in cell-free systems. The authors also found that the preincubation of neutrophils with catechins before stimulation with PMA enhanced the inhibitory activity of all compounds [103], which also counts in favor of this hypothesis.

A comparison of the inhibitory activity of polyphenols showed that catechol-containing flavonoids and hesperetin, which have a methoxy substituent at the 4′-position, effectively inhibited intracellular CL. Lipophilic hesperetin was more effective than hydrophilic eriodictyol, taxifolin, and quercetin. Earlier, Ribeiro and coauthors demonstrated that the analogs of luteolin methylated at the 3′- or 4′-position of the B ring (chrysoeriol and diosmetin, respectively) are more effective inhibitors of the LCL of PMA-stimulated neutrophils (total ROS production) than luteolin. In addition, using aminophenyl fluorescein as a probe, it was shown that these compounds are more effective in decreasing the intracellular production of HOCl than luteolin [98]. Based on these data, the authors suggested that the presence of a vicinal (*ortho-*) hydroxy-methoxy arrangement in the aromatic ring may enhance the inhibitory potency. The authors also noted that the methylation of the hydroxy groups led to an increase in the lipophilicity of the compounds, which influences their ability to incorporate into the lipids of cell membranes and pass through them and, therefore, their intracellular accessibility. At the same time, the authors showed that an increase in lipophilicity is not the only factor that determines the high activity of the compounds [98,99]. For instance, an analog of luteolin that contains two methoxy groups in the B-ring was significantly less active than chrysoeriol, diosmetin, and luteolin itself. According to our data, lipophilic naringenin, which bears only one hydroxyl group in the B-ring, was less active than the other compounds studied, which is in line with the data of Ribeiro [98]. It is also important that DfTf, which contains a strongly hydrophilic carboxyl group in the structure and is the most hydrophilic among all compounds tested, has no effect on intracellular ROS production.

At the same time, it was found that DfTf, along with catechol-containing flavonoids and hesperetin, significantly decreased the extracellular level of ROS produced by PMA-stimulated neutrophils. These compounds exerted similar inhibitory effects. The results obtained agree well with the literature data [98,99,103,104], in particular, with the data of Drábiková and coauthors, who studied possible mechanisms involved in the inhibitory effects of coumarin derivatives on the activity of neutrophils [104]. The authors demonstrated that a more lipophilic compound significantly inhibited both the extracellular and intracellular ROS production, whereas less lipophilic coumarin decreased significantly only the extracellular production of ROS and had little effect on intracellular ROS production. As mentioned above, Vuotto and coauthors showed that lipophilic catechins inhibited the extracellular CL activity of PMA-stimulated neutrophils to the same extent as their more hydrophilic precursor, catechin. The latter, in turn, was significantly inferior to its derivatives in the inhibition of the intracellular CL response [103].

**Figure 7:**
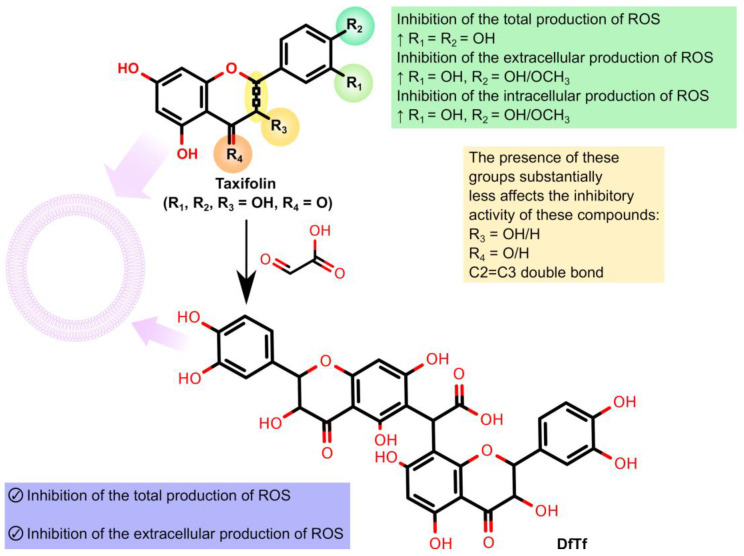
Relationship between the chemical structure and inhibitory activity of the polyphenols being tested against ROS production by neutrophils activated with PMA.

Thus, our results suggest that the catechol-containing polyphenols significantly inhibited the total, intra, and extracellular production of ROS, which is consistent with the literature data [98,99]. Compounds containing a methoxy group at the 3′- or 4′-position retained high inhibitory activity, which sometimes exceeded that of unsubstituted polyphenols. Summarized data on the structure–inhibitory activity relationships of the polyphenols tested are given in Figure 7.

The following points should be noted. In cell-free systems, DfTf showed the highest hydrogen peroxide-scavenging activity (Table 1), indicating that DfTf possesses high antioxidant activity against ROS present in the aqueous phase. Catechol-containing flavonoids were less effective than DfTf but more effective than hesperetin. However, these compounds exerted comparable inhibitory effects on the extracellular ROS production by neutrophils. It is also noteworthy that hydrophobic hesperetin (logP 2.6), which predominantly accumulates in the hydrophobic phase, showed inhibitory activity comparable with that of DfTf (logP 1.13), a hydrophilic compound, which is present in the aqueous phase in larger quantities and scavenges ROS more effectively than hesperetin. Thus, the data obtained suggest that polyphenols are able to both scavenge ROS and interfere with the processes involved in ROS production by neutrophils.

Presumably, the inhibitory effect of polyphenols on the oxidative burst of neutrophils is brought about through various mechanisms [102,105]. As was mentioned above, they can scavenge ROS and bind transition metal ions, which, under certain conditions, leads to the inhibition of the Fenton reaction [106,107,108,109,110] and protection of cells against the damage mediated by metal ions [110,111,112].

Polyphenols are able to modulate the activity of signaling pathways involved in the generation of ROS. For instance, it was shown that polyphenols such as resveratrol, curcumin, and quercetin inhibited the PMA-induced activation of protein kinase C [105,113,114]. Quercetin and 2′,5′-dihydroxy-2-furfurylchalcone inhibited the activation of phospholipase D induced by fMLP [115,116]. Resveratrol inhibited the activation of PLD induced by C5a [117].

There is substantial evidence indicating that polyphenols can inhibit the key enzymes involved in the production of ROS by neutrophils [118,119,120,121]. In particular, among natural polyphenols, the inhibitors of NADPH oxidase were identified [122,123,124,125], e.g., apocynin, a (mono) methoxy-substituted catechol, and myricitrin, a glycosyloxyflavone [122,123]. It is remarkable that apocynin seems to be a prodrug. To produce an inhibitory effect, apocynin should be converted by peroxidase to its more active form, a dimer [124,126,127,128]. Some phenolic acid derivatives also act as inhibitors of NOX2 [129]. It was noted that phenolic acid derivatives exhibiting inhibitory activity had increased hydrophobicity. It was suggested that NADPH oxidase as a membrane protein is more easily accessible for these lipophilic compounds [129].

In addition, it was shown that some flavonoids, in particular, taxifolin, hesperetin, and naringenin, inhibited the activity of endothelial NADPH oxidase [119]. It is interesting that catechol-containing flavonoids such as quercetin, catechin, epicatechin, and luteolin did not exhibit NADPH oxidase-inhibitory activity, whereas the methylation or the loss of one of the hydroxyl groups from the catechol moiety enhanced the inhibitory potency [119]. For instance, methylated derivatives of epicatechin (3′- and 4′-O-methyl epicatechin), in contrast to the parent flavonoid, showed inhibitory activity. In addition, the (-)-epicatechin dimer procyanidin B2 and its O-methylated derivate effectively inhibited NADPH oxidase activity [119]. Thus, these data suggest that epicatechin, similarly to apocynin, may be a prodrug, which is converted to its active forms, a dimer and methylated derivatives by myeloperoxidase (MPO) and catechol-O-methyltransferase, respectively [118,119].

Another key enzyme involved in ROS production is MPO. It catalyzes a reaction between hydrogen peroxide (H_2_O_2_) and chloride anion (Cl^−^) to form a strong oxidant, hypochlorous acid, which subsequently can chlorinate biomolecules, including lipids, proteins, and nucleic acids [130]. It was shown that flavonoids can act as substrates and inhibitors of MPO [118,130,131,132]. In particular, flavonoids are excellent one-electron donors, which are able to reactivate the (pseudo-) halogenating activity of MPO after the H_2_O_2_-mediated inactivation, indicating that they can compete with chloride for MPO [131]. Epicatechin, eriodictyol, and catechin exhibited a high reactivation potential, whereas luteolin and taxifolin displayed a low capacity for reactivation. On the whole, the highest effect was found for those compounds that contain hydroxyl groups at the 3′, 4′, 5, and 7 positions [131].

A strong inhibitory effect of flavonoids such as quercetin, kaempferol, and myricetin on the activity of MPO was demonstrated by Shiba and coauthors [130]. These compounds contain in their structure hydroxyl groups at the 3, 5, and 4′ positions and double bond C2=C3 [130]. At the same time, fisetin, which lacks the hydroxyl group at the 5 position, luteolin, which lacks the hydroxyl group at the 3 position, and taxifolin, which lacks the C2=C3 double bond, were less effective than flavonoids mentioned above. Thus, it can be assumed that these structural features are required for the manifestation of the inhibitory effect [130]. The authors also found that the inhibitory effect of the flavonoids depends on their hydrophobicity [130].

One further flavonoid acting as a competitive substrate and effective inhibitor of MPO is EGCG [132,133]. It was found that EGCG, similar to quercetin, effectively inhibited MPO- (or neutrophil-)mediated HOCl formation in vitro and protected endothelial cells from neutrophil-induced injury [120,121,132]. In addition, dietary quercetin significantly inhibited aortic endothelial dysfunction in diabetic mice in vivo. This compound simultaneously suppressed the expression and activity of vascular MPO [121].

Thus, polyphenols are multitarget agents against oxidative stress whose effects on cells depend on their structure and environment.

One more important thing should also be noted. In contrast to the flavonoids tested herein, DfTf effectively decreased only the extracellular level of ROS generated by PMA-stimulated neutrophils. However, its action outside the cells was comparable with that of some effective inhibitors of the oxidative burst of neutrophils, such as quercetin, taxifolin, eriodictyol, catechin, and hesperetin. In addition, DfTf showed the highest hydrogen peroxide-scavenging activity in cell-free systems. The data obtained suggest that DfTf can protect cells against the toxic effect of extracellular ROS. Taking this into account, it can be assumed that DfTf may protect against oxidative stress-related pathological conditions or diseases when excessive extracellular production of ROS leads to cell and tissue damage [75]. The beneficial effects of polyphenols on such kind of diseases/pathological conditions have also been reported in the literature [134,135,136]. In particular, polyphenols are considered promising agents for treating inflammatory diseases of the GIT. As was mentioned above, it is in this compartment that the concentration of polyphenols might achieve their maximum values [135,137,138]. At the same time, DfTf did not affect the production of intracellular ROS, suggesting that this compound does not influence the processes mediated by these reactive species. Intracellular ROS are known to be involved in microbial killing and act as signaling molecules; therefore, their production is necessary for an effective innate immune response [75,139].

Thus, DfTf may be considered a potential agent capable of minimizing the toxic effects of extracellular ROS without affecting intracellular ROS production and associated processes. The identification of these agents and the signaling pathways that specifically control extracellular, but not intracellular, ROS production is of great therapeutic interest [74,75].

## 4. Materials and Methods

### 4.1. Chemicals

Quercetin, catechin, eriodictyol, hesperetin, naringenin, 5-amino-2,3-dihydro-1,4- phthalazinedione (luminol), 4-aminophthalhydrazide (isoluminol), HRP (150 U/mg), catalase (2000 U/mg), SOD (2050 U/mg), Hoechst 33342, and propidium iodide were purchased from Sigma-Aldrich (St. Louis, MO, USA). Taxifolin was kindly provided by Flavit (Pushchino, Russia). Hydrogen peroxide was obtained from Reakhim (Moscow, Russia). Phosphate buffered saline (PBS), ficoll-urografin 1.077/1.119, and Hanks’ solution were purchased from Paneco (Moscow, Russia). All reagents were of analytical grade purity. Water used for the preparation of solutions was purified using a Milli-Q system (Millipore, Burlington, MA, USA).

### 4.2. Synthesis of DfTf

DfTf was synthesized as described previously [47]. The synthesis methods for this compound are presented in the Supplementary Materials (Materials and Methods S1).

### 4.3. Hydrogen Peroxide-Scavenging Activity

The hydrogen peroxide-scavenging activity of polyphenols was estimated by isoluminol-dependent chemiluminescence (ICL) as described previously [47]. Briefly, the reaction mixtures contained NaH_2_PO_4_/Na_2_HPO_4_ buffer (20 mM, pH 7.4), isoluminol (100 µM), HRP (0.25 U), hydrogen peroxide (10 µM), and polyphenols at different concentrations. The concentrations of naringenin were 0.6, 1.3, 2.5, 8.0, 15.0, 31.0, 63.0, and 125 μM. The concentrations of taxifolin, quercetin, catechin, eriodictyol, hesperetin, and DfTf were 0.1, 0.2, 0.4, 0.6, 0.9, 1.3, 2.0, 3.0, and 5.0 μM. Hydrogen peroxide was added to the mixtures immediately prior to the registration of a signal. ICL signals were recorded until ICL returned to the baseline. The integral ICL response was calculated as a sum of ICL values recorded during the measurements. Changes in ICL caused by polyphenols were estimated relative to the control ICL values by the equation: ∫ICL = (∫polyphenol/∫control) × 100%, where ∫ICL is the integral ICL, %; ∫polyphenol is the integral ICL in the presence of polyphenols; ∫control is the integral ICL without the addition of polyphenols. Measurements were carried out on a Tecan Infinite F200 microplate reader (Grödig, Austria) in 96-well plates (Greiner, Kremsmunster, Austria) at 37 °C.

### 4.4. Isolation of Neutrophils

Neutrophils were isolated as described previously [76,140]. Briefly, peripheral blood from rats was collected into tubes containing 100 μL of heparin (5000 U/mL). Erythrocytes were lysed by mixing heparinized blood with ice-cold distilled water in a ratio of 1:2 for 20 s. Then, the osmolarity was quickly restored by adding 2 × PBS, and the mixture was centrifuged for 5 min at 180× *g* at 4 °C. The pellet was washed with PBS. The resulting leukocyte-rich fraction was layered on a gradient of Ficoll-urografin 1.077/1.119. After centrifugation for 15 min at 180× *g* at 4 °C, a fraction of cells located at the Ficoll-urografin 1.077/1.119 interface was collected and washed twice with PBS. Then, the cells were resuspended in Hanks’ solution (pH 7.2). The content of neutrophils in the fraction was no less than 96%.

### 4.5. Cell Viability

Neutrophils (5 × 10^4^/well) were incubated with polyphenols at different concentrations (the maximal concentration was 50 μM) in 96-well microplates for 3 h. After that, the cells were stained with Hoechst 33342 (5 μg/mL) and propidium iodide (10 μg/mL). Hoechst 33342 stained live and dead cell nuclei, whereas propidium iodide stained dead cell nuclei. Then, the cells were examined using the ImageXpress Micro XL (Molecular Devices, Sunnyvale, CA, USA). The number of viable cells was more than 96% after incubation with polyphenols at the maximal concentration tested (Supplementary Figure S3).

### 4.6. Production of ROS

The production of ROS by neutrophils was estimated by the method of enhanced chemiluminescence (CL), as described previously [76]. The total ROS production was evaluated using luminol as a dye, which is able to penetrate through cell membranes and react with ROS generated both intra and extracellularly.

Neutrophils (5 × 10^4^/well) were incubated in 96-well plates (Greiner, Kremsmunster, Austria) for 10 min at 37 °C in Hanks’ medium containing polyphenol at different concentrations and luminol (100 μM). The concentrations of naringenin were 0.18, 0.35, 0.70, 1.5, 2.5, 4.8, 12, 20, and 40 μM. The concentrations of taxifolin, quercetin, catechin, eriodictyol, and hesperetin were 0.18, 0.35, 0.70, 1.5, and 2.5 μM. The concentrations of DfTf were 2.5, 4.8, 12, 20, and 40 μM. After incubation, the cells were stimulated with PMA (2.8 × 10^−7^ M).

To detect the intracellular production of ROS, the membrane-impermeable enzymes SOD and catalase, which remove extracellularly released O_2_^•−^ and H_2_O_2_, were added to the reaction mixture. Neutrophils (5 × 10^4^/well) were incubated for 10 min at 37 °C in Hanks’ medium containing polyphenol, luminol (100 μM), SOD (50 U/mL), and catalase (2000 U/mL) [72]. The concentration of naringenin was 20 μM. The concentration of taxifolin, quercetin, catechin, eriodictyol, and hesperetin was 1.5 μM. The concentrations of DfTf were 2.5, 5.0, and 20 μM. After incubation, the cells were stimulated with PMA (2.8 × 10^−7^ M).

The extracellular production of ROS was evaluated using the membrane-impermeable isomer of luminol isoluminol as a dye. Neutrophils (5 × 10^4^/well) were incubated for 10 min at 37 °C in Hanks’ medium containing polyphenol at different concentrations, isoluminol (100 μM), and HRP (6 U/mL). The concentrations of naringenin were 10 and 20 μM. The concentrations of taxifolin, quercetin, catechin, eriodictyol, and hesperetin were 0.75, 1.5, and 2.25 μM. The concentrations of DfTf were 1.0, 1.5, 2.2, 5.0, and 10 μM. After incubation, the cells were stimulated with PMA (2.8 × 10^−7^ M).

CL signals were recorded at 37 °C for 3.5 h. The integral CL response was calculated as the sum of CL values recorded during the measurements. The effect of polyphenols on CL was estimated using the following formula: ∫CL = (∫polyphenol/∫control) × %, where ∫CL is the integral CL, %; ∫polyphenol is the integral CL in the presence of polyphenols; ∫control is the integral CL without the addition of polyphenols. IC_50_ values were subsequently calculated from dose-response curves using the Hill equation. The measurements were performed in 96-well plates (Greiner, Kremsmunster, Austria) at 37 °C using a Tecan Infinite 200 multiplate reader (Grödig, Austria).

### 4.7. Statistical Analysis

All experiments were performed at least three times in triplicate. The data were expressed as the mean ± standard deviation. Differences between groups of data were analyzed by one-way analysis of variance (ANOVA) followed by Tukey’s test (GraphPad Prism 9.0.0, GraphPad Software Inc., San Diego, CA, USA). Differences with a *p*-value < 0.05 were considered statistically significant.

## 5. Conclusions

The results obtained indicate that all compounds decreased the total amount of ROS generated by PMA-stimulated neutrophils. Flavonoids exhibited comparable inhibitory activity both inside and outside cells. A product of the condensation of taxifolin with glyoxylic acid had no effect on intracellular production of ROS but significantly decreased extracellular ROS production. The extracellular inhibitory activity of the product was comparable with that of effective inhibitors of the oxidative stress found among flavonoids (catechol-containing compounds and hesperetin). Thus, the condensation of flavonoids with toxic carbonyl compounds may lead not only to the elimination of the latter but also to the formation of products that are capable of significantly inhibiting the oxidative burst of neutrophils. The data also suggest that, in the course of these reactions, the influence of polyphenolic fractions on the functions of the cells may change. In general, the results of the study provide a better understanding of the impact of polyphenols, including those formed during condensation reactions, on human health. In addition, these results reveal a structure–activity relationship of these polyphenols and may be useful in the search for new therapeutic agents against oxidative stress-related pathological conditions or diseases when the excessive extracellular production of ROS leads to cell and tissue damage.

## Figures and Tables

**Table 1:** Concentrations of polyphenols necessary for the 50% inhibition of chemiluminescence in a cell-free system.

Polyphenol	IC_50_, μM ^1^	
	Isoluminol-HRP-H_2_O_2_	Luminol-HRP-H_2_O_2_ [53]
DfTf	0.44 ± 0.04 *	0.48 ± 0.04 *
eriodictyol	0.57 ± 0.04	0.73 ± 0.05
quercetin	0.60 ± 0.02	0.75 ± 0.05
taxifolin	0.64 ± 0.02	0.76 ± 0.05
catechin	0.66 ± 0.05	0.93 ± 0.09
hesperetin	1.19 ± 0.05 *	2.17 ± 0.15 *
naringenin	18.68 ± 2.67 *	34.2 ± 4.32 *

^1^ The values are the means ± standard deviation. * Significantly different from other compounds, *p* < 0.001.

**Table 2:** Concentrations of polyphenols necessary for the 50% inhibition of luminol-dependent chemiluminescence of neutrophils stimulated with PMA.

Polyphenol	IC_50_, μM ^1^
eriodictyol	0.59 ± 0.06
quercetin	0.39 ± 0.03 #
taxifolin	0.51 ± 0.08
catechin	0.49 ± 0.07
hesperetin	0.94 ± 0.03 *
naringenin	26.6 ± 4.54 *

^1^ The values are the means ± standard deviation. * Significantly different from other compounds, *p* < 0.0001, # Differences are significant compared with eriodictyol, *p* < 0.002.

**Table 3:** Concentrations of polyphenols necessary for the 50% inhibition of isoluminol-dependent chemiluminescence of neutrophils stimulated with PMA.

Polyphenol	IC_50_, μM ^1^
DfTf	1.70 ± 0.21
eriodictyol	1.74 ± 0.25
quercetin	1.43 ± 0.10
taxifolin	1.77 ± 0.20
catechin	0.84 ± 0.27
hesperetin	1.23 ± 0.14
naringenin	14.3 ± 2.17 *

^1^ The values are the means ± standard deviation. * Significantly different from other compounds, *p* < 0.0001.

**Table 4:** Lipophilicity of the polyphenols being tested.

Polyphenol	LogP
DfTf	1.13 ± 0.02 [47]
taxifolin	1.57 ± 0.03 [94]
quercetin	1.82 ± 0.32 [95]
catechin	1.80 [96]
eriodictyol	2.27 ± 0.02 [95]
	2.02 [97]
hesperetin	2.6 [97]
naringenin	2.30 ± 0.18 [94]
	2.60 ± 0.03 [95]
	2.52 [97]

## Data Availability

The data presented in this study are available upon request from the corresponding author.

## Supplementary Materials

The following supporting information can be downloaded at https://www.mdpi.com/article/10.3390/ijms242015068/s1.
