# Supplementary material for: A Comparative Study of the Inhibitory Effect of Some Flavonoids and a Conjugate of Taxifolin with Glyoxylic Acid on the Oxidative Burst of Neutrophils
Source: Int J Mol Sci. 2023 Oct 11;24(20):15068. doi: 10.3390/ijms242015068 (PMC10606308; doi:10.3390/ijms242015068)
Supplement: Supplementary file 1 [file ijms-24-15068-s001.zip › ijms-2622175-supplementary.pdf]

International Journal of *Molecular Sciences*

Supplementary Materials

A Comparative Study of the Inhibitory Effect of Some Flavonoids and a Conjugate of Taxifolin with Glyoxylic Acid on the Oxidative Burst of Neutrophils

Victoria S. Shubina*, Victoria I. Kozina, and Yuri V. Shatalin*

Institute of Theoretical and Experimental Biophysics, Russian Academy of Sciences, Institutskaya 3, 142290 Pushchino, Russia

* Correspondence: shubinavs@iteb.pushchino.ru (V.S.S.); shatalinyuv@iteb.pushchino.ru (Y.V.S.)

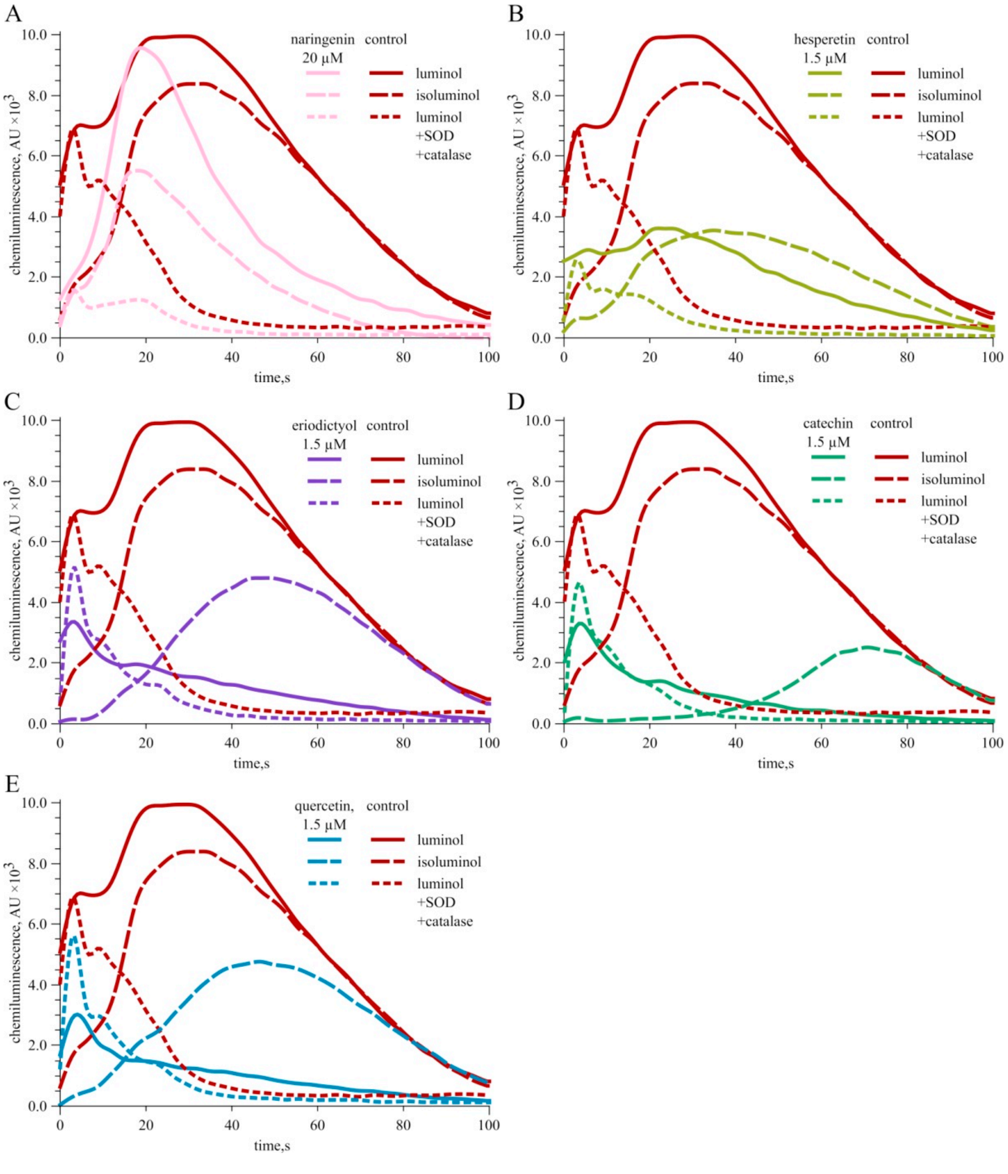

Figure S1. Effect of polyphenols on chemiluminescence of neutrophils stimulated with PMA: (A) naringenin; (B) hesperetin; (C) eriodictyol; (D) catechin; (E) quercetin.

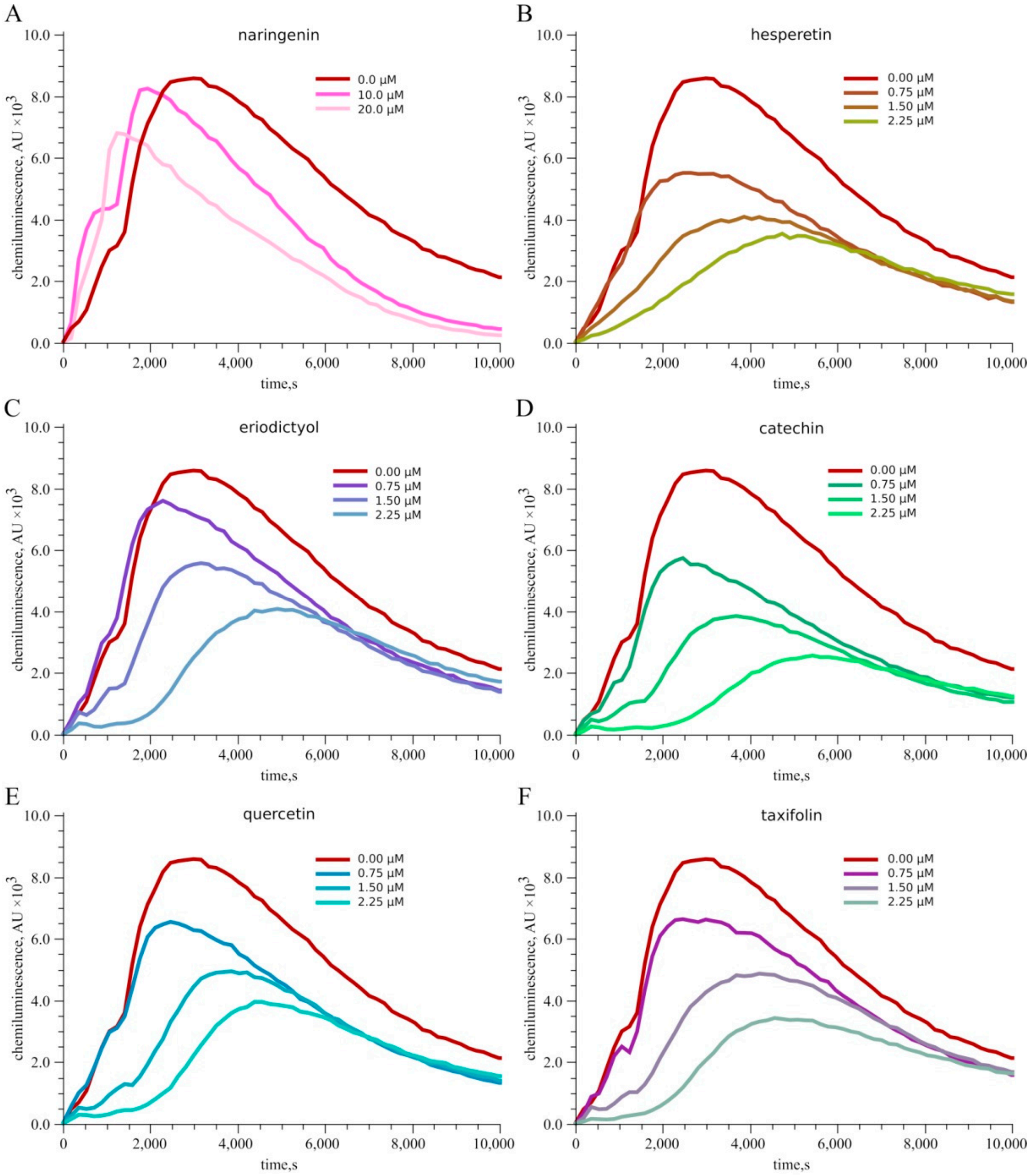

Figure S2: Time courses of isoluminol-dependent chemiluminescence of neutrophils in the control and in the presence of different concentrations of flavonoids.

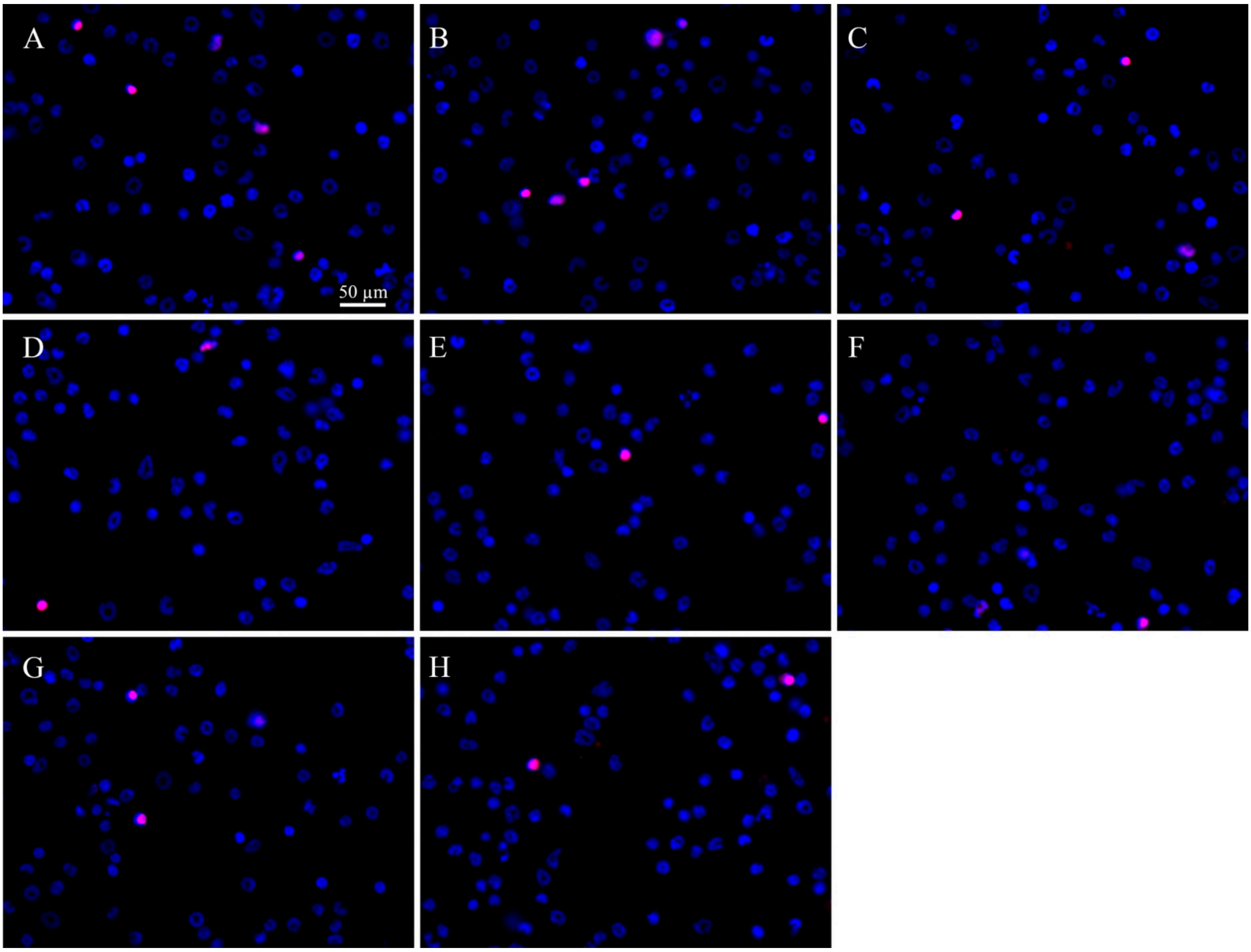

Figure S3: Representative fluorescence microscopy images of neutrophils after incubation with polyphenols (50 µM) for 3 h: (A) control; (B) DfTf; (C) eriodictyol; (D) quercetin; (E) taxifolin; (F) catechin; (G) hesperetin; (H) naringenin. The cells were stained with Hoechst 33342 (blue) and propidium iodide (red). Hoechst 33342 stained live and dead cell nuclei, whereas propidium iodide stained dead cell nuclei. Scale bar: 50 µm.

Materials and Methods S1: the synthesis of the condensation product of taxifolin with glyoxylic acid (DfTf).

The condensation product of taxifolin with glyoxylic acid (DfTf) was synthesized as described previously [1]. Briefly, a mixture of taxifolin (608 mg), glyoxylic acid (148 mg), concentrated hydrochloric acid (20 µl) and dry tetrahydrofuran (5 ml) was refluxed for 2 h. The reaction progress was monitored by thin-layer chromatography. After completion of the reaction, the solvent was evaporated under vacuum. The condensation product was isolated in the form of a sodium salt, washed by heated ethanol and dried under vacuum. The structural features of the resulting product were determined by NMR spectroscopy [1].

References

1. Shubina, V.S.; Shatalin, Y.V. Antioxidant and Iron-Chelating Properties of Taxifolin and Its Condensation Product with Glyoxylic Acid. J Food Sci Technol 2017, 54, 1467–1475, doi:10.1007/s13197-017-2573-0.
